# Therapeutic efficacy and safety of *Botulinum Toxin A Therapy* in *Trigeminal Neuralgia*: a systematic review and meta-analysis of randomized controlled trials

**DOI:** 10.1186/s10194-016-0651-8

**Published:** 2016-07-05

**Authors:** Mostafa Ebraheem Morra, Ahmed Elgebaly, Ahmed Elmaraezy, Adham M. Khalil, Ahmed M. A. Altibi, Tran Le-Huy Vu, Mostafa Reda Mostafa, Nguyen Tien Huy, Kenji Hirayama

**Affiliations:** Faculty of Medicine, Al Azhar University, Cairo, 11884 Egypt; Faculty of Medicine, Zagazig University, Zagazig, 44519 Egypt; Faculty of Medicine, University of Jordan, Amman, 11942 Jordan; University of California, Los Angeles, Los Angeles, CA 90095 USA; Faculty of Medicine, Tanta University, Tanta, 31527 Egypt; Department of Clinical Product Development, Institute of Tropical Medicine (NEKKEN), Leading Graduate School Program, and Graduate School of Biomedical Sciences, Nagasaki University, 1-12-4 Sakamoto, Nagasaki, 852-8523 Japan; Center for Molecular Bio-Medicine, University of Medicine and Pharmacy, 217 Hong Bang, District 5, Ho Chi Minh City, Ho Chi Minh 70000 Viet Nam; Department of Immunogenetics, Institute of Tropical Medicine (NEKKEN), Leading Graduate School Program, and Graduate School of Biomedical Sciences, Nagasaki University, 1-12-4 Sakamoto, Nagasaki, 852-8523 Japan

**Keywords:** Botulinum, BTX-A, Trigeminal neuralgia, Clinical trials, Systematic review, Meta-analysis

## Abstract

**Background:**

Several different interventions have been examined to alleviate pain and reduce frequency of trigeminal neuralgia (TN) paroxysms. However, some patients continue to have persistent or recurrent painful attacks. Using a systematic review and meta-analysis approach, we aimed to synthesize evidence from published randomized controlled trials (RCTs) regarding safety and efficacy of botulinum toxin type A (BTX-A) as a possible emerging choice of treatment for TN.

**Methods:**

We conducted an electronic search in 10 databases/electronic search engines to access relevant publications. All articles in all languages reporting RCTs on the efficacy and safety of BTX-A in the treatment of TN were included for systematic review and meta-analysis.

**Results:**

A total of four RCTs (n = 178) were identified for final meta-analysis. The overall effect favored BTX-A versus placebo in terms of proportion of responders (risk ratio RR = 2.87, 95 % confidence interval CI [1.76, 4.69], p <0.0001) with no significant detected heterogeneity (p = 0.31; I^2^ = 4 %). Paroxysms frequency per day was significantly lower for BTX-A group (mean difference MD = -29.79, 95 % CI [-38.50,–21.08], p <0.00001) with no significant heterogeneity (p = 0.21; I^2^ = 36 %).

**Conclusion:**

Despite limited data, our results suggest that BTX-A may be an effective and safe treatment option for patients with TN. Further larger and well-designed RCTs are encouraged to translate these findings into better clinical outcome and better quality of life for TN patients.

**Electronic supplementary material:**

The online version of this article (doi:10.1186/s10194-016-0651-8) contains supplementary material, which is available to authorized users.

## Introduction

Trigeminal neuralgia (TN) is a characteristic pain along the distribution of one or more branches of the trigeminal nerve. The pain is usually unilateral and described as severe, sharp, and stabbing electric shock-like pain. Consequently, quality of life of TN patients is profoundly worsened due to impairment of daily life activities, thus patients are at a high risk of depression and other psychiatric disorders [1–3]. Epidemiological studies showed that approximately 4 to 28.9/100,000 persons worldwide experience TN [4, 5]. Moreover, a recent large retrospective cohort study reported increased risk of depression and anxiety disorders in TN patients [6]. To date, the mechanisms underlying the pathogenesis of TN are still controversial; however, the microvascular compression is the most common hypothesis [7, 8].

Recently, therapeutic and surgical approaches have been evolved to alleviate the neuropathic pain and improve quality of life in TN patients. Oral antiepileptic drugs, including carbamazepine, remain the first line of treatment [9]. Yet, 25–50 % of cases became refractory to the drug therapy [10]. Invasive operations such as neurovascular decompression [11], gamma knife radiosurgery [12], partial sensory rhizotomy, and percutaneous radiofrequency thermo-coagulation [13] may be required in some intractable cases. Then again, Surgical interventions occasionally effect severe and often untreatable complications that might be even worse than the primary TN [14]. Moreover, a study reported recurrence of the pain in about half of the patients within 2 years of percutaneous radiofrequency rhizotomy [15]. Recently, a cross specialty management program for TN patients was implemented in a tertiary referral center for headache and pain [16]. The program involved collaboration between neurologists, neuroradiologists, and neurosurgeons and showed promising in terms of feasibility and utility in management of TN [16].

In light of the evidence currently available, it seems fair to argue that there is an overwhelming need for developing much safer, better tolerated, and efficacious treatment for TN. Botulinum toxin type A (BTX-A) is a neurotoxin derived from *Clostridium botulinum* [17]. It acts via inhibiting release of acetylcholine at neuromuscular junctions, causing relaxation of the muscle [18, 19]. BTX-A has been shown to be a promising option for treatment of headache [20, 21]. It was further approved by the US Food and Drug Administration (FDA) for prevention of headache in adults suffering from chronic migraine.

In 2002, Micheli and colleagues [22] reported successful relief of twitching and pain in a patient with TN-associated hemifacial spasm. Since then, several clinical trials have been investigating the safety and efficacy of BTX-A in TN. The drug showed favorable effects in many reports. Having said that, the majority of studies were at a high risk of bias, aside from the small sample size included in each trial. This necessitates a confirmatory evidence to be released asserting the notion that BTX-A may be safe and effective option for management of TN.

The aim of this study is to provide a clear-cut evidence regarding safety and efficacy of botulinum toxin type A in trigeminal neuralgia from published randomized controlled trials in a systematic review and meta-analysis approach.

## Methods

### Search Strategy

The study strictly followed the recommendation of Preferred Reporting Items for Systematic Reviews and Meta-Analyses (PRISMA) statement (Additional file 1: Table S1). During September and October, we established the protocol and registered our study with PROSPERO, University of York (CRD42015026861). Ten electronic search engines/libraries were systematically searched for relevant publications, including PubMed, Scopus, Web of Science, Google Scholar, Virtual Health Library (VHL), WHO Global Health Library (GHL), ClinicalTrials, POPLINE, System for Information on Grey Literature in Europe (SIGLE), and the New York Academy of Medicine (NYAM). Except for Google Scholar, the search term for all other libraries was as follows: (botulinum OR onabotulinumtoxinA OR onabotulinum OR botox OR botulinus) AND ((trigeminal neuralgia) OR prosopalgia OR (Fothergill's disease) OR (tic doloureux)). For Google Scholar, we used the advanced setting in which “trigeminal neuralgia” was filled in “with all of the words” and “botulinum botulinus botox onabotulinum toxin A onabotulinum” for “with at least one of the words”. Additionally, we conducted a manual search by reviewing the citations within the included publications and reviewing the related references presented in PubMed.

### Selection criteria and title and abstract screening

Search results from the ten aforementioned search engines/libraries were imported into Endnote X7 (Thompson Reuter, CA, USA) for duplicates deletion. Two reviewers independently screened the references using the predetermined eligibility criteria. The inclusion criteria were: (i) randomized-controlled trials (RCTs) reporting efficacy and safety of botulinum toxin in treatment of trigeminal neuralgia and (ii) no restriction on language, area, publication year, and age of patients. The exclusion criteria were: (i) unreliably extracted data; (ii) over-lapped data sets; (iii) book chapters, abstract articles only, conference papers, reviews, theses, and posters; and (iv) *in vitro* or animal studies. Any different decision in screening step was discussed between two reviewers to reach a consensus. Consultation from supervisor (NTH and KH) was acquired if necessary. The full texts of included references were then retrieved through the Library of Nagasaki University, and full text screening was subsequently conducted to identify relevant references for data extraction.

### Outcome measures

All patients’ outcomes were considered to analyze the efficacy and safety of botulinum toxin in the treatment of trigeminal neuralgia: (1) proportion of respondents – defined as patients with >50 % reduction in mean pain score from baseline to endpoint; (2) mean paroxysms frequency per day; (3) mean visual analog scale (VAS) score at the end of follow up; and (4) adverse events and complications of BTX A treatment.

### Data extraction

The standardized template was developed through a pilot extraction with the two most relevant references. Two researchers then independently extracted the data into the template. Extracted data included: authors, publication year, journal, country of authors, country of patients, source of BTX-A, active drug types and their doses, control types and their doses, number and site of injection, characteristics of patients (gender and age), duration of treatment, duration of follow up, year of neuralgia, affected branch, surgical procedures, efficacy point, proportion of responders, relapse, visual analog scale, number of paroxysms per day, quality of life, anxiety scale, and depression scale .

### Statistical method

Statistical analysis was carried out using RevMan version 5.3 (The Cochrane Collaboration, Oxford, United Kingdom). Continuous data were pooled as mean difference (MD), while pooled risk ratio (RR) was combined for dichotomous data using Mantel-Haenszel (M-H) method. Fixed effect model was adopted in all analyses. We performed a sensitivity analysis to: (1) investigate the effect of the model assumed on the overall effect size, by running the analysis under the random effects model and observing if a difference exists (2) explore the effect of the study quality on the summary effect estimate, by omitting studies rated as "high risk of bias" for both random sequence generation and allocation concealment. Heterogeneity was assessed by visual inspection of the forest plots and measured by I-square and Chi-square tests. P-values reported in this paper are not adjusted for multiplicity; however, adequate randomization and blinding was achieved by most included studies, ensuring appropriate control for confounders. *P*-value < 0.05 was considered statistically significant.

### Quality assessment

The methodological quality of each RCT was independently assessed by two reviewers using "The Cochrane Collaboration's tool for assessing risk of bias". It is a two-part tool, addressing seven specific domains, including: sequence generation, allocation concealment, blinding of participants and personnel, blinding of outcome assessment, incomplete outcome data, selective outcome reporting, and other sources of bias. The judgment of each reviewer on each domain was categorized as ‘low risk,’ ‘high risk,’ or ‘unclear risk’ of bias. Any disagreement was resolved by discussion between two reviewers and by consultation from a supervisor to reach a consensus.

## Results

### Literature search

**Fig. 1:**
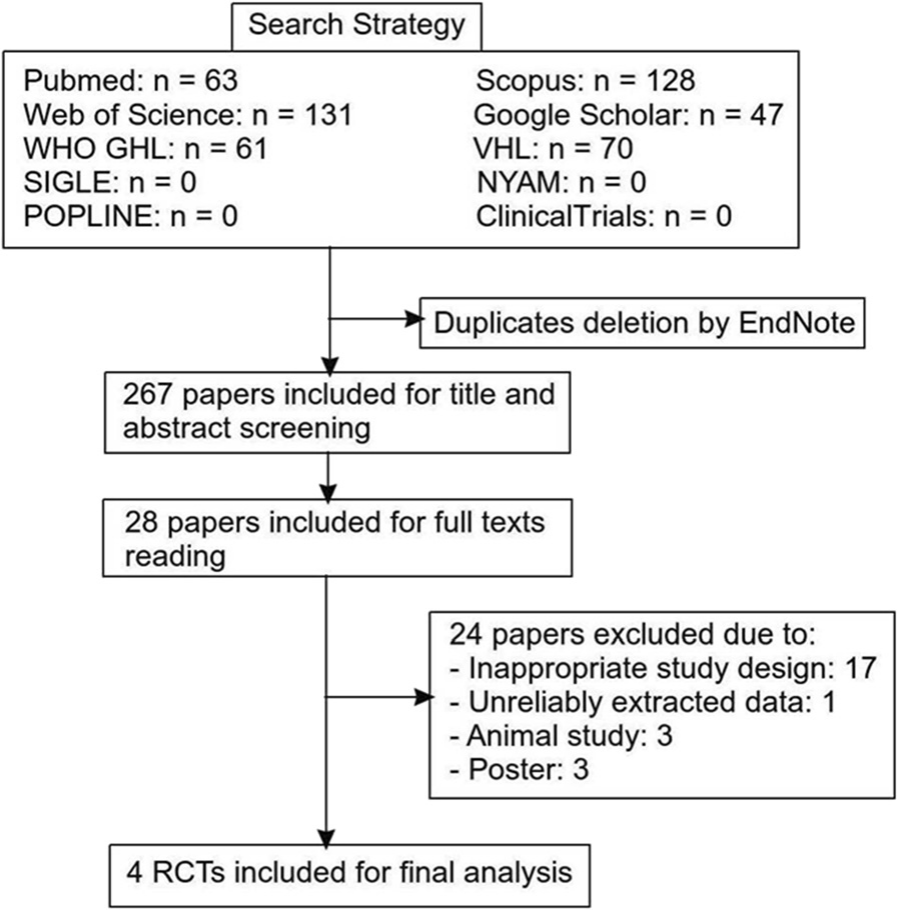
PRISMA flow diagram of studies’ screening and selection

A total number of 267 articles were retrieved from six search engines/libraries (Fig. 1). SIGLE, POPLINE, NYAM, and ClinicalTrials generated no results. After the initial title and abstract screening of the 267 articles, 28 articles were selected for full-text reading. Two independent reviewers performed the full-text screening after which 24 articles were excluded due to: (1) inappropriate study design; (2) unreliably extracted data; (3) in vitro or animal study; and (4) posters. Finally, a total of four unique RCTs, with a total of 178 *TN* patients, were included for data extraction and final analysis.

### Study characteristics

The characteristics of four RCTs included in the final analysis were summarized in Table 1. A total of 178 patients (99 in the BTX-A group and 79 in the placebo control group) were included from four trials. The mean age for both case and control groups in three RCTs [23–25] was ranging from 58.1 to 64.5 for case group and from 58 to 66 for control group. There was no significant difference between BTX-A and placebo groups in terms of frequency of attacks and pain severity before treatment as reported in each included study. The follow-up period varied from 8 to 12 weeks except for no report from study of Zhang et al. The mean duration of the effect was also ranging between 8 to 12 weeks in two trials [23, 25].

**Table 1:** Baseline characteristics of included RCTs

Author	No. of patients			Mean age (year)			Mean duration before treatment (year)		Frequency of attacks per day before treatment (SD)		Pain severity before treatment, VAS Mean (SD)			Mean follow up (week)		Mean duration of effect (week)	
	cases		control	cases		control	cases	control	cases	control	cases		control	cases	control	cases	control
Zhang et al. [24]	25 Group1: 25U	28 Group2: 75U	27	58.16	62.64	58.41	ND	ND	ND	ND	6.24 (2.13)	7.18 (2.21)	6.96 (1.97)	ND	ND	ND	ND
Shehata et al. [26]	10		10	ND		ND	ND	ND	36.7 (3.13)	39.2 (3.05)	8.3		8.5	12	12	ND	ND
Wu et al. [23]	20		22	59.14		58	ND	ND	21.71 (22.68)	20.53 (10.38)	7.05 (2.03)		6.88 (2.25)	12	12	12	12
Zúñiga et al. [25]	16		20	64.5		66.06	6.2	5.2	29.1 (31.36)	31.06 (31.62)	8.85		8.19	8	8	8	8

*ND: no data*

*RCT: randomized controlled trials*

The amount of BTX-A injected, the route of injection, the number of injections, and the injection site varied among studies. The amount of BTX-A injected ranged from a minimum of 25 U in study of Zhang et al. [24] to a maximum of 100 U in study of Shehata et al. [26]. In addition, 50 U and 75 U of BTX-A were used in studies of Zúñiga et al. [25] and Wu et al. [23], respectively. The administration of BTX-A included subcutaneous or intradermal routes. The details of the injection protocol in each trial are summarized in Table 2.

**Table 2:** Injection protocol of BTX-A

Author	Type of BTX-A	Source of BTX-A	Amount of BTX-A	Injection sites	No. of injection	Rout of injection	Blinding
Zhang et al. [24]	Botulinum toxin type A (BTX-A)	Lanzhou Bioproduction Institute, Lanzhou, China	Group 1:25u Group 2:75u	Dermatome and/or mucosa	20	Intradermal and submucosal	Double blind
Shehata et al. [26]	Botulinum toxin type A (Botox®)	Not reported	100u	Trigger zones	1	SC	Double blind
Wu et al. [23]	BTX-A (100U of Clostridium botulinum type-A neurotoxin complex, 5 mg gelatin, 25 mg dextran, and 25 mg saccharose)	Lanzhou Biological Products Institute, China	75u	Trigger zones	15	Intradermal	Double blind
Zúñiga et al. [25]	Onabotulinum toxin A (Botox)	Not reported	50u	Various sites, 1 cm apart from one another	1	SC	Double blind

*SC* Subcutaneous

### Quality assessment

**Fig. 2:**
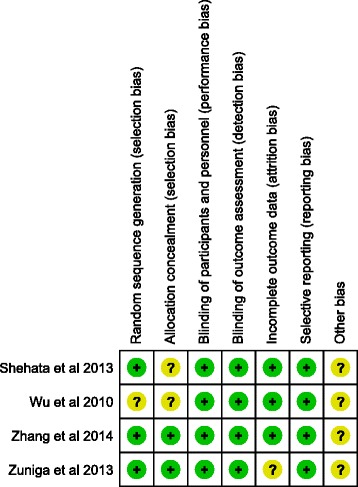
Cochrane bias assessment of included studies

The quality of included studies ranged from medium to high as illustrated in Fig. 2. Authors’ judgments with justifications are shown in (Additional file 2: Table S2).

### Therapeutic efficacy

Three patients’ therapeutic outcomes include: (1) proportion of respondents – defined as patients with >50 % reduction in mean pain score from baseline to endpoint; (2) mean paroxysms frequency per day; and (3) mean visual analog scale (VAS) score at the end of follow up.

**Fig. 3:**
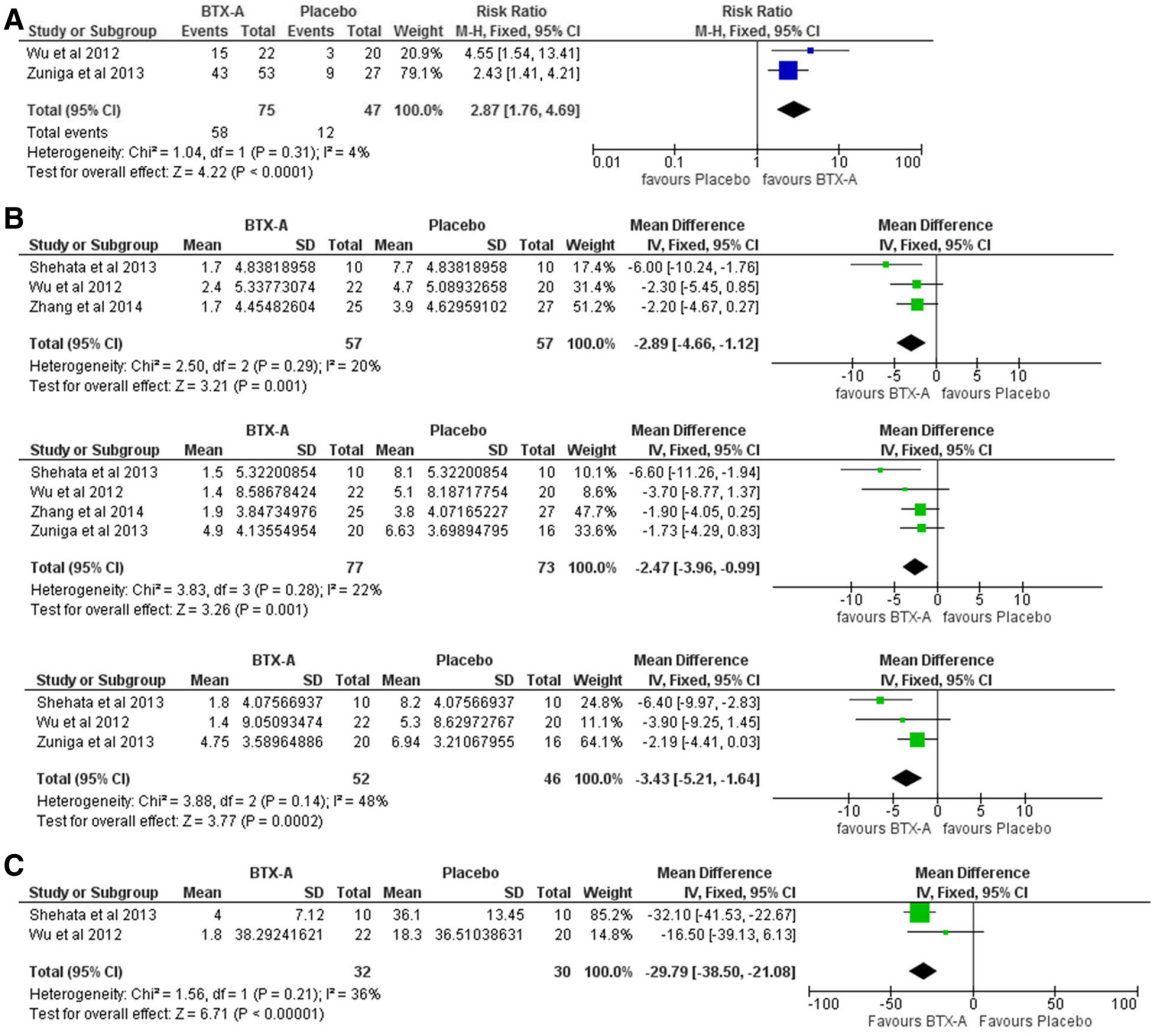
**A** Forest plots of relative risk (RR) for proportion of respondents comparing BTX A with placebo. M-H: Mantel-Haenzel, CI: confidence interval. **B** Forest plots of mean difference in VAS score comparing BTX A with placebo at the end of follow up (A), 1 month (B), 2 month (C), and 3 month (D). VAS: Visual analogue scale, MD: Mean difference, M-H: Mantel-Haenzel, CI: confidence interval. **C** Forest plots of mean difference in number of paroxysm comparing BTX A with placebo. MD: Mean difference, M-H: Mantel-Haenzel, CI: confidence interval

The overall effect favored BTX-A compared with placebo in terms of proportion of responders (RR = 2.87, 95 % CI [1.76, 4.69], P < 0.0001) with no significant heterogeneity detected (P = 0.31; I^2^ = 4 %) (Fig. 3a). Mean VAS score was significantly lower for BTX-A group at the end of the first month (MD = -2.89, 95 % CI [-4.66,–1.12], P = 0.001), at the end of the 2^nd^ month (MD = -2.47, 95 % CI [-3.96,–0.99], P = 0.001), and at the end of the 3^rd^ month of follow up (MD = -3.43, 95 % CI [-5.21,–1.64], P = 0.0002) with no significant heterogeneity detected for all endpoints (Fig. 3b). Mean paroxysms frequency per day was significantly lower for BTX-A group (MD = -29.79, 95 % CI [-38.50,–21.08], P < 0.00001) with no significant heterogeneity (P = 0.21; I^2^ = 36 %) (Fig. 3c).

### Adverse events

Regarding safety of BTX-A, there were two reported injection-related side effects: facial asymmetry and edema/hematoma at the site of injection, and both of which were generally tolerated and transitory in nature. The overall occurrence of facial asymmetry in BTX-A group ranged from 2-5 patients while it ranged between 1–2 for edema/hematoma. Facial asymmetry recovered within 5–7 weeks while edema/hematoma resolved within 5–6 days. None of the controlled patients developed facial asymmetry or edema/hematoma except two patients in Shehata *et al*. [26] and one patient in Wu et al. [23]. The adverse events were fully summarized in Table 3.

**Table 3:** Adverse events for all patients in included RCTs

Author/reference	Facial asymmetry			Edema/hematoma at injection area		
	Exposed	Control	Disappearance	Exposed	Control	Disappearance
Zhang et al. [24]	3	0	6 weeks	2	0	5 days
Shehata et al. [26]	4	0	not reported	1	2	not reported
Wu et al [23]	5	0	7 weeks	2	1	7 days
Zúñiga et al [25]	2	0	not reported	2	0	not reported

### Additional analysis

**Fig. 4:**
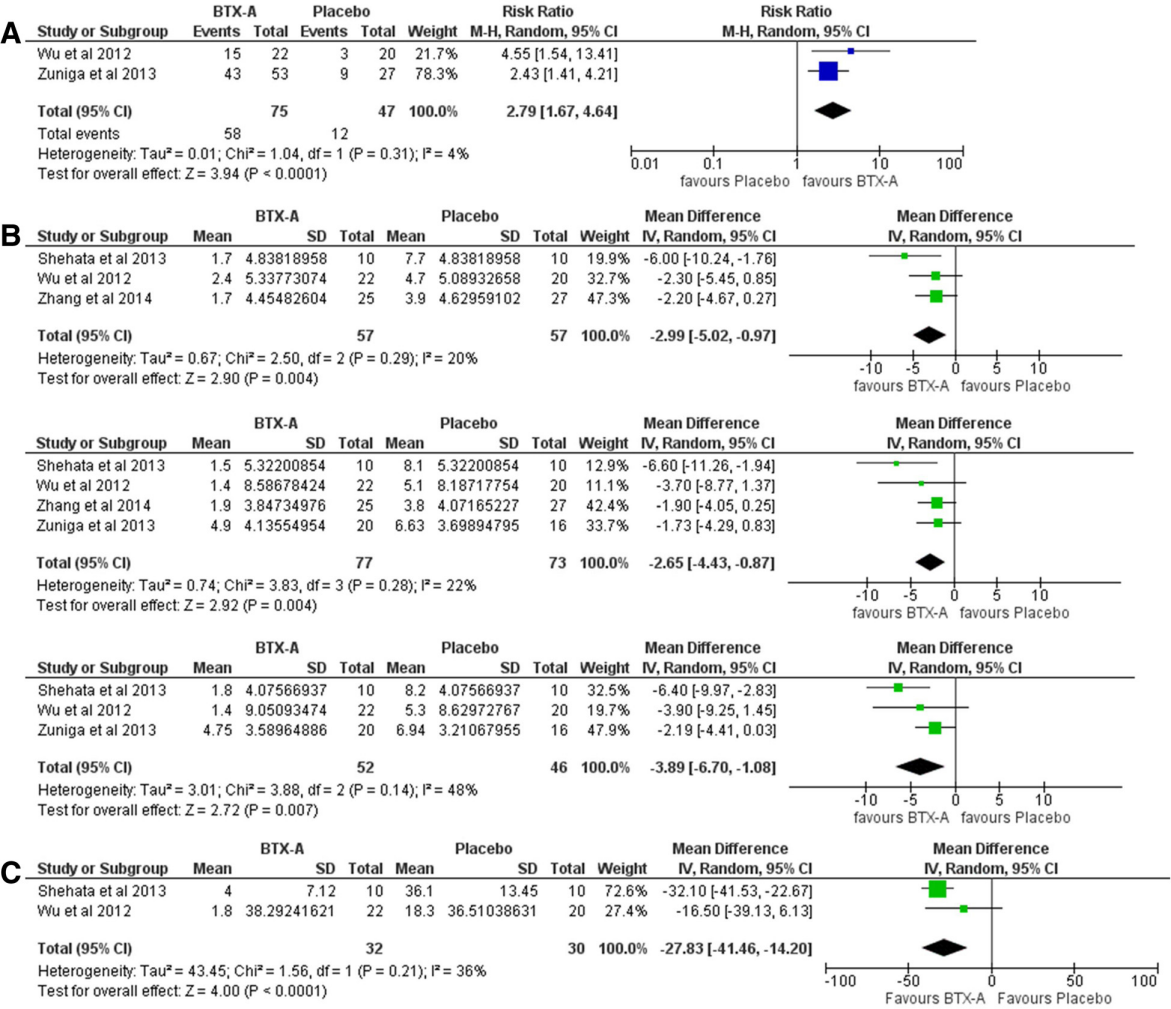
Forest plots of Sensitivity analysis (random effects model)

**Fig. 5:**
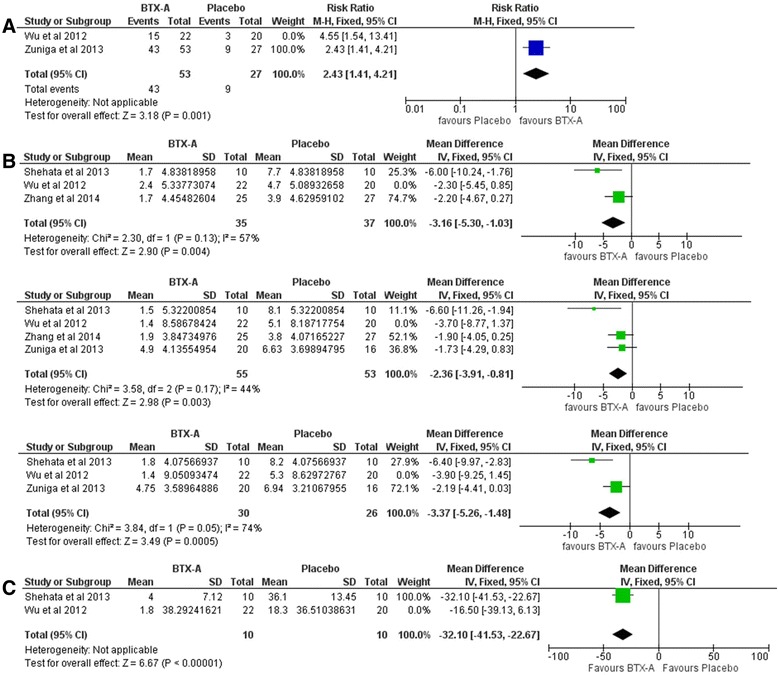
Forest plots of Sensitivity analysis (removing one study at a time)

Only one study [23] had both sequence generation and allocation concealment rating of "high risk of bias". Omitting this study from the analysis, slightly changed the effect estimate and CI but did not have a serious impact on the findings (Fig. 4). Moreover, the overall effect estimate did not differ significantly for any of the outcomes on using the random effects model (Fig. 5). This in turn adds to the robustness of the results.

## Discussion

Treatment of TN continues to present a clinical appeal to both patients and health care providers. Medications (such as antiepileptic drugs) were poorly tolerated. They effected central nervous system adverse events in a substantial proportion of patients [27]. As such, refractory cases were approached by alternative surgical interventions, which yielded marked improvement in about 63–94 % of cases [28]. Nonetheless, serious complications of invasive surgery (including aseptic meningitis and hearing loss) rendered further sufferings to TN patients [28].

Thus safer, well tolerated, and more effective alternative is imperative for TN. The evidence from this systematic review suggests that botulinum toxin type A (BTX-A) has a clinically significant benefit in treatment of trigeminal neuralgia when compared to placebo in terms of proportion of responders, the mean paroxysms frequency per day, and the VAS score at the end of follow up. These overall outcomes consistently favored BTX-A compared with placebo across studies.

The duration of effect for BTX-A seems to be of relatively long duration (at least 3 months). In Wu et al. [23] trial, BTX-A was effective within a median follow-up duration of 90 days. Also, Zuniga et al. [25] showed that BTX-A was effective during the 3 months follow-up period. However, a conclusion cannot be made on how long the effect of BTX-A lasts, and how often recurrent injections are needed. This raises the need for further studies with extended follow-up duration that implement chronic pain measurement scales. A point of variation is the dosage of BTX-A; the used dosage of BTX-A in the analyzed RCTs ranged from 25 to 100 U. Zhang et al. [24] found that lower dose (25 U) and higher dose (75 U) were similar in efficacy on short-term assessment. Moreover, a dramatic response to BTX-A was also reported in an open-label trial [29] at a much lower dose of BTX-A (6.45–9.11 U).

The treatment mechanism of BTX-A in TN remains unclear. Previous preclinical studies suggested that it acts locally or on the trigeminal ganglia [30, 31]. Conversely, Matak et al. reported a central antinociceptive effect of BTX-A in a rat model of TN [32, 33]. Wu et al. recently asserted this notion and proved that peripherally applied BTX-A exerts antinociceptive function via reducing central sensitization and inhibiting high expression of nociceptors [34].

It is noteworthy to mention that Yong Hu et al. [35] approached the same topic with our study, but they mostly included open-label trials, which potentially weakened their conclusion. Furthermore, by combining one randomized, double-blind, placebo control trial with five open-label trials, the previous study possibly introduced some heterogeneity into their review.

One limitation of the current systematic review was the small number of trials that investigated the safety and efficacy of BTX-A in trigeminal neuralgia. Therefore, we cannot explore the publication bias using Egger's test for funnel plot asymmetry, we rather searched clinical trial registries and grey literature and found only two protocols of ongoing studies. No completed unpublished studies were retrieved. Missing data (mean baseline age in Shehata et al. and length of follow up in Zhang et al.) could not be used to adjust for possible biases in the aforementioned studies. More double-blinded RCTs are required to elaborate more on (1) optimal dose of BTX-A, (2) optimal duration of treatment, and (3) optimal route of administration. In addition, further research is needed to explore more on the mechanism of action of BTX-A to relieve pain in trigeminal neuralgia.

## Conclusions

In conclusion, our systematic review suggests that BTX-A is a promising alternative treatment option that might spare the need for surgical interventions for refractory cases of trigeminal neuralgia in the future. Evidence from larger and well-designed RCTs is still required to assert upon these findings.

## Article highlights

- ➢ RCTs reporting the efficacy and safety of BTX-A in the treatment of TN were included for systematic review and meta-analysis.
- ➢ BTX-A is an effective and safe treatment option for patients with trigeminal neuralgia.
- ➢ No serious adverse events or systemic reaction of BTX-A have been reported.

## Additional files

Additional file 1:
**Table S1.** The PRISMA checklist. (DOCX 28 kb) Additional file 2:
**Table S2.** Risk of bias with author judgments with justifications. (DOCX 17 kb)
