# Supplementary material for: Therapeutic efficacy and safety of Botulinum Toxin A Therapy in Trigeminal Neuralgia: a systematic review and meta-analysis of randomized controlled trials
Source: J Headache Pain. 2016 Jul 5;17(1):63. doi: 10.1186/s10194-016-0651-8 (PMC4932020; doi:10.1186/s10194-016-0651-8)
Supplement: Additional file 2: — Table S2. Risk of bias with author judgments with justifications. (DOCX 17 kb) [file 10194_2016_651_MOESM2_ESM.docx]

**Additional file 2. Risk of bias with author judgments with justifications**

| ROB of: Zuniga et al 2013(21)  Bias | Authors' judgement | Support for judgement |
| --- | --- | --- |
| Random sequence generation (selection bias) | Low | "Candidates interested in the study signed an informed consent of agreement to participate in the latter and were randomized by central allocation concealment," |
| Allocation concealment (selection bias) | Low | "Candidates interested in the study signed an informed consent of agreement to participate in the latter and were randomized by central allocation concealment," |
| Blinding of participants and personnel (performance bias) | Low | Double-blinded |
| Blinding of outcome assessment (detection bias) | Low | Double-blinded |
| Incomplete outcome data (attrition bias) | Unclear | No mention of N of patients who randomized and who completed the study |
| Selective reporting (reporting bias) | Low |  |
| Other bias | Unclear | No mention for study funding |

| ROB of: Wu et al. 2013(20) | Authors' judgement | Support for judgement |
| --- | --- | --- |
| Random sequence generation (selection bias) | Unclear | No mention of the method. |
| Allocation concealment (selection bias) | Unclear | No mention of the method. |
| Blinding of participants and personnel (performance bias) | Low | Double-blinded |
| Blinding of outcome assessment (detection bias) | Low | Double-blinded |
| Incomplete outcome data (attrition bias) | Low | About 90% of randomized patients completed the study |
| Selective reporting (reporting bias) | Low |  |
| Other bias | Low |  |

| ROB of Shehata et al 2013(25) | Authors' judgment | Support for judgment |
| --- | --- | --- |
| Random sequence generation (selection bias) | Low | Adequate- "Computer-generated randomization" |
| Allocation concealment (selection bias) | Unclear | No mention of the method of concealment |
| Blinding of participants and personnel (performance bias) | Low | "Double-Blinded" |
| Blinding of outcome assessment (detection bias) | Low | "Double-Blinded" |
| Incomplete outcome data (attrition bias) | Low | All randomized patients were included into final analysis |
| Selective reporting (reporting bias) | Low |  |
| Other bias | Unclear | No mention of the funding |

| ROB of Zhang et al2013(22) | Authors' judgment | Support for judgment |
| --- | --- | --- |
| Random sequence generation (selection bias) | Low | "computer-generated randomization list" |
| Allocation concealment (selection bias) | Low | "Randomization data were kept strictly confidential, accessible only by authorized persons. The data was locked and verified and unblended only when the trial was completed." |
| Blinding of participants and personnel (performance bias) | Low | Double-blinded |
| Blinding of outcome assessment (detection bias) | Low | Double-blinded |
| Incomplete outcome data (attrition bias) | Low | 95% of randomized patients enter the analysis. |
| Selective reporting (reporting bias) | Low |  |
| Other bias | Low |  |
